# The Large-Scale Preparation and Optical Properties of MoS_2_/WS_2_ Vertical Hetero-Junction

**DOI:** 10.3390/molecules25081857

**Published:** 2020-04-17

**Authors:** Tao Han, Hongxia Liu, Shulong Wang, Shupeng Chen, Kun Yang

**Affiliations:** Key Laboratory for Wide-Bandgap Semiconductor Materials and Devices of Education, the School of Microelectronics, Xidian University, Xi’an 710071, China; taohan373@gmail.com (T.H.); spchen@xidian.edu.cn (S.C.); kuny2019@163.com (K.Y.)

**Keywords:** MoS_2_/WS_2_ vertical hetero-junction, optical properties, Raman spectrum, PL spectrum, AFM, SEM

## Abstract

A variety of hetero-junctions can be constructed to form the basic structural units in the different optoelectronic devices, such as the photo-detectors, solar cells, sensors and light-emitting diodes. In our research, the large-area high-quality MoS_2_/WS_2_ vertical hetero-junction are prepared by the two-step atmospheric pressure chemical vapor deposition (APCVD) methods and the dry transfer method, and the corresponding optimal reaction conditions of MoS_2_/WS_2_ vertical hetero-junction are obtained. The morphology, composition and optical properties of MoS_2_/WS_2_ vertical hetero-junction are systematically characterized by the optical microscopy, Raman spectroscopy, photoluminescence spectroscopy, atomic force microscopy and the field emission scanning electron microscopy. Compared to the mechanical transfer method, the MoS_2_/WS_2_ vertical hetero-junction sample obtained by the APCVD and dry transfer methods have lower impurity content, cleaner interfaces and tighter interlayer coupling. Besides, the strong interlayer coupling and effective interlayer charge transfer of MoS_2_/WS_2_ vertical hetero-junction are also further studied. The photoluminescence intensity of MoS_2_/WS_2_ vertical hetero-junction is significantly reduced compared to the single MoS_2_ or WS_2_ material. In general, this research can help to achieve the large-scale preparation of various Van der Waals hetero-junctions, which can lay the foundation for the new application of optoelectronic devices.

## 1. Introduction

The electronic chips integration increase with the development of modern semiconductor technology, and the volume of devices is becoming smaller, with the preparation processes becoming more complex [1,2,3]. The size of conventional Si-based CMOS devices has approached the limit, so researchers turn their attention to the two-dimensional transition metal dichalcogenides (2D TMDs) materials with single atomic layers. However, there are some limitations in the application of the single two-dimensional materials. The single h-BN material cannot be used in the devices alone, due to its wide band gap [4]. Additionally, the exposed BP is easily oxidized in the air, which would lead to the degradation of its performance [5]. As we all know, different two-dimensional materials have different energy band structures and band gaps. To further investigate the intrinsic properties of two dimensional materials and expand the application fields, the two dimensional hetero-junction materials can concentrate the advantages of two materials together to achieve the precise adjustment of the band gap, and it can also finely regulate the physical properties of the single materials. The advantage of Van der Waals (VDWs) atomic level hetero-junction has the convenience of modular combination, compared with the conventional hetero-junction. In other words, the atomic layer materials could be combined to prepare the hetero-junctions at will, which does not need to consider the lattice matching [6,7]. The corresponding hetero-junctions can be formed by stacking these atomic layer materials, which would show some properties not available in the single material. Besides, it can also achieve complementary properties.

The 2D TMD materials have large band gaps of a two-dimensional structure from the visible light to the near-infrared spectrum, adjustable carrier mobility, and electronic and optical characteristics related to the layer number [8,9]. It is these excellent characteristics that make them show great potential in the application of optoelectronic devices, such as the photo detectors [10], and the light emitting diodes [11]. The combination of MoS_2_ and WS_2_ films can effectively adjust the optical and electrical properties of MoS_2_/WS_2_ vertical hetero-junction through interlayer coupling, which would result in effective charge transfer and band structure reorganization. Meanwhile, they have a rich phenomenon of physics and electronics, which can also provide new opportunities for the design of novel electronic devices and photonic devices. In the previous reports, the mechanical stripping and chemical vapor deposition methods are usually used for the controlled and scalable production of the transition metal sulfides and the large-scale synthesis of TMDs with ideal architecture [12,13]. The mechanical exfoliation and directional transfer methods are the most widely used to study the VDWs hetero-structures, but the successful accumulation is accidental, and the methods have lower yield and efficiency [14]. Although some progress has been made in the preparation of TMD hetero-structures by the chemical vapor deposition method (CVD), conventional methods can usually only produce the lateral structures and not vertical hetero-structures. Therefore, there is still a huge challenge in using the existing exfoliation or CVD growth processes to achieve the scalable production of TMDs hetero-junctions with well-controlled structures.

The VDWs hetero-junction can be formed by using mechanical transfer technology to stack the different 2D materials, which has limited stacking orientation control. Compared to the mechanical transfer method, the direct growth of MX_2_ hetero-structure by CVD method not only has inherent scalability, but also has a cleaner interface and tighter interlayer coupling [15]. In this paper, the two-step atmospheric pressure chemical vapor deposition (APCVD) and dry transfer methods are used to epitaxially grow WS_2_ on MoS_2_, which can prepare the MoS_2_/WS_2_ vertical hetero-junction with its clean interface and strong interlayer coupling. The optimal processes’ reaction conditions of MoS_2_/WS_2_ vertical hetero-junction can also be obtained. Meanwhile, the morphology, composition and optical properties of MoS_2_/WS_2_ vertical hetero-junction are systematically characterized by optical microscopy, Raman spectroscopy, photoluminescence (PL) spectroscopy, atomic force microscopy, and field emission scanning electron microscopy. The results shown in this paper can provide effective guidance for future photonic devices, solar cells, photo-detectors, modulators, and storage devices [16].

## 2. The Introduction of Experiment

### 2.1. The Growth Experiments of MoS_2_ or WS_2_ by APCVD

**Figure 1:**
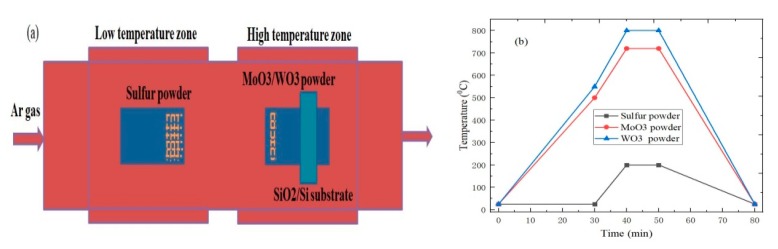
(**a**) Schematic diagram of MoS_2_ or WS_2_ materials growth experiment by atmospheric pressure chemical vapor deposition (APCVD); (**b**) The temperature change diagram of MoS_2_ or WS_2_ materials experiment process by APCVD.

The sulfur powder, tungsten trioxide, and molybdenum trioxide are respectively used as the sulfur source, tungsten source, and molybdenum source in the MoS_2_ or WS_2_ materials growth experiment, and the MoS_2_ and WS_2_ materials are respectively grown on SiO_2_/Si substrates by the atmospheric pressure chemical vapor deposition (APCVD) method. The following are the specific growth processes of single MoS_2_ or WS_2_ materials [17]. First, the SiO_2_/Si substrates are sequentially put into the absolute ethanol, acetone, and deionized water for 10 minutes, and then washed with the deionized water. Next, the first quartz boat with 0.1 g sulfur powder is put into the low temperature zone of the dual-temperature zone tube furnace. At the same time, the second quartz boat, equipped with 2 mg molybdenum source or tungsten source and the cleaned SiO_2_/Si substrate, is placed in the high temperature zone of the double temperature zone tube furnace, as shown in Figure 1a. Then, the tube furnace is evacuated to the vacuum state (1 Pa) when the vacuum pump is turned on, and then the high purity argon gas is used to fill the entire quartz tube chamber to reach the normal pressure state (100 KPa). The above operation is repeated three times to eliminate the air of the quartz tube. Subsequently, the heating program of the tube furnace is opened, and the growth temperatures of MoS_2_ and WS_2_ are respectively set to 720 °C and 800 °C due to the different sublimation points. The temperature of MoO_3_ powder increased from 25 °C to 720 °C, which was completed with a rate of 15.8 °C/min for 30 min and in turn 22 °C/min for 10 min. Meanwhile, the temperature of WO_3_ powder was increased from 25 °C to 800 °C, and successively heated at a rate of 17.5 °C/min for 30 min and 25 °C/min for 10 min. After the MoO_3_ and WO_3_ powders completed the first-stage heating, the temperature of sulfur powder increased from 25 °C to 200 °C in 10 min. Besides, the heating time remained unchanged for 10 min under the growth condition, and the flow rate of shielding gas is 35 sccm during the reaction. Finally, the heating program was turned off when the growth of single MoS_2_ or WS_2_ materials was completed, and the corresponding samples were taken out when the tube furnace was cooled to room temperature.

### 2.2. The Preparation Experiment of MoS_2_/WS_2_ Vertical Heterojunction

Meanwhile, the preparation processes of the MoS_2_/WS_2_ vertical hetero-junction by the dry transfer method are also described in previous work [18]. Based on the previous MoS_2_ and WS_2_ materials prepared by APCVD, the polymethyl methacrylate (PMMA) liquid was spin-coated on the WS_2_ at a speed of 4000 r/s, and annealed at 150 °C for 30 min. Additionally, the PMMA film can be immersed in the NaOH solution (Lingping Chemical Glass Instrument Co., Ltd., Guangzhou, China, 0.1 Mol/L). Then, the PMMA film with WS_2_ is floated and transferred to another SiO_2_/Si substrate, by using the transfer platform under the condition of CCD monitoring, which would overlap with the MoS_2_. Finally, the PMMA film is dissolved with acetone, and the MoS_2_/WS_2_ vertical hetero-junction can be obtained.

### 2.3. The Characterization of MoS_2_/WS_2_ Vertical Heterojunction

The Raman spectrum and PL spectrum of the MoS_2_/WS_2_ vertical hetero-junction are systematically analyzed by the LabRam HR Evolution Raman microscopy, equipped with the 532 nm laser under the 1mW laser power, and the laser spot size is 1μm. The high-resolution images can be obtained by using the 50× objective lens, 1800 lines/mm grating and the 500 nm imaging steps [19]. At the same time, the field emission scanning electron microscope (FESEM) and atomic force microscope (AFM) are also used to characterize the surface morphology and thickness of the MoS_2_/WS_2_ vertical hetero-junction.

## 3. The Analysis of Characterization Results

**Figure 2:**
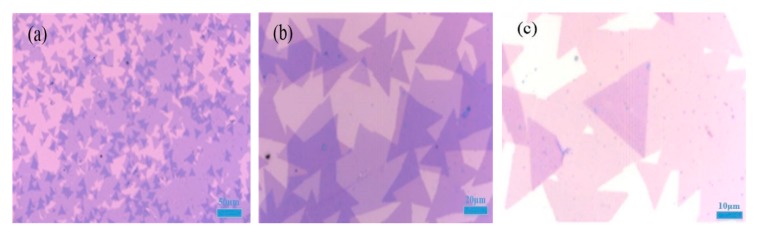
Optical microscopy images of MoS_2_/WS_2_ vertical hetero-junction at different magnifications (**a**) 10×; (**b**) 50×; (**c**) 100× objectives.

In order to obtain better MoS_2_/WS_2_ vertical hetero-junction materials, we first need to grow the high-quality large-area single MoS_2_ or WS_2_ materials by APCVD. Under the optimal growth conditions, the gas concentration and nucleation density of single MoS_2_ or WS_2_ materials in the tube furnace can achieve the maximum values [20]. The MoS_2_/WS_2_ double-layer vertical hetero-junction can be prepared by the two-step APCVD method and the dry transfer processes. At the same time, the MoS_2_/WS_2_ vertical hetero-junction samples are systematically characterized by optical microscopy, Raman spectroscopy, PL spectroscopy, AFM and FESEM. In addition, the surface of WS_2_, MoS_2_, and MoS_2_/WS_2_ vertical hetero-junction samples are all uniform triangles with a size of several tens of microns after the corresponding growth reaction, and the color contrast between the samples and SiO_2_/Si substrate is relatively obvious, which can be distinguished by the optical microscope, as shown in Figure 2. 

The MoS_2_/WS_2_ vertical hetero-junction on SiO_2_/Si substrate can also be observed by the FESEM. It can be found that the size of single WS_2_ or MoS_2_ materials are respectively 20 μm and 30 μm, and the contrast between single WS_2_ or MoS_2_ materials and SiO_2_/Si substrate is very uniform. 

**Figure 3:**
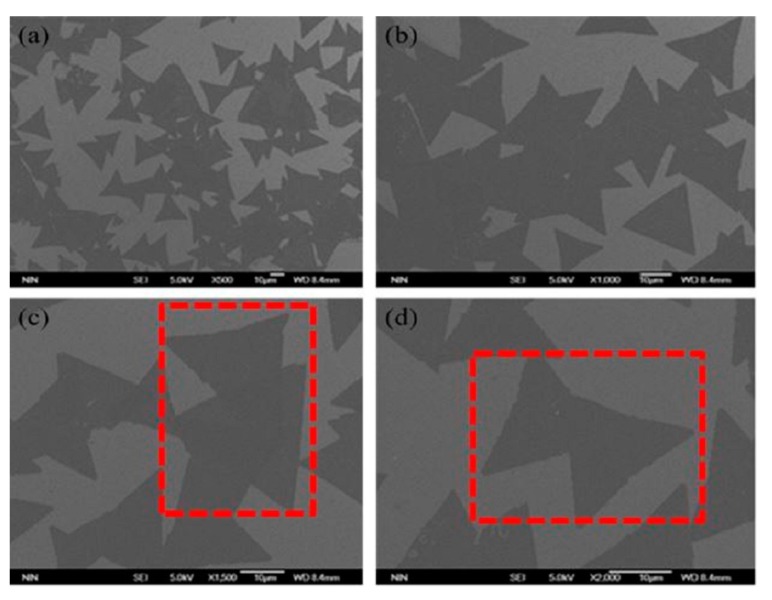
Field emission scanning electron microscope (FESEM) characterization of MoS_2_/WS_2_ vertical hetero-junction at different magnifications (**a**) 500×; (**b**) 1000×; (**c**) 1500×; (**d**) 2000×.

The reason the overlapping parts are not mixed together to form the WS_2_-MoS_2_ lateral hetero-junction is that we have adopted the two-step growth process. The first step is that the WoO_3_ powder forms the WS_2_ crystals during the high temperature (800 °C) reduction and vulcanization process, and the second step is that the MoO_3_ forms MoS_2_ crystals during the low temperature (720 °C) reduction and vulcanization process. Besides, the PMMA-assisted transfer method is used to transfer the monolayer WS_2_ to the monolayer MoS_2_ material, and the top WS_2_ layer has only 0° and 60° orientation relative to the bottom MoS_2_ layer in the red dotted box, which respectively correspond to A-A and A-B stacks [21,22], as shown in Figure 3. Therefore, unlike the traditional CVD method, the injection of Mo into Wo_3_ to form the lateral MoS_2_-WS_2_ mixture is effectively prevented. 

**Figure 4:**
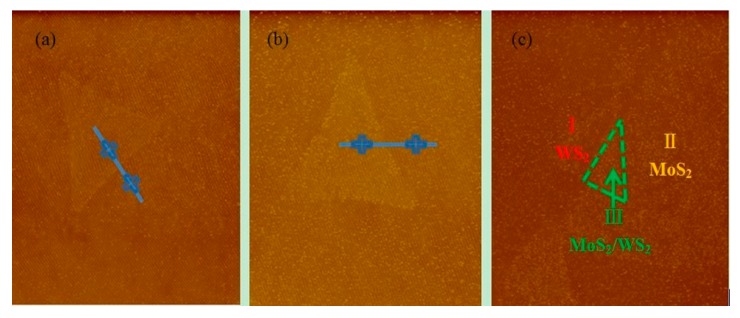
Atomic force microscope (AFM) image of (**a**) MoS_2_; (**b**) WS_2_; (**c**) MoS_2_/WS_2_ vertical hetero-junction.

Figure 4 shows the AFM images of MoS_2_, WS_2_, and MoS_2_/WS_2_ vertical hetero-junction. It can be found by observing the surface morphology and thickness that the thickness of the MoS_2_/WS_2_ vertical hetero-junction is about 1.7 nm, as shown in red mark region III. In order to further determine the thickness of single MoS_2_ and WS_2_ materials, the thickness of region II MoS_2_ and region I WS_2_ are respectively measured as 0.8 nm and 0.9 nm, which indicates the existence of monolayer WS_2_ and monolayer MoS_2_, and the lateral dimension is about 20–30 μm.

## 4. Introduction and Analysis of the Spectrum Characteristics

Raman spectrum and PL spectrum have become the effective way to detect and identify the optical properties and layer number of MoS_2_, WS_2_, and MoS_2_/WS_2_ vertical hetero-junction grown by the APCVD and dry transfer methods. The corresponding test characterizations require constant temperature and humidity, which are performed in an ultra-clean room environment.

### 4.1. The Spectrum Characterization of Monolayer WS_2_

**Figure 5:**
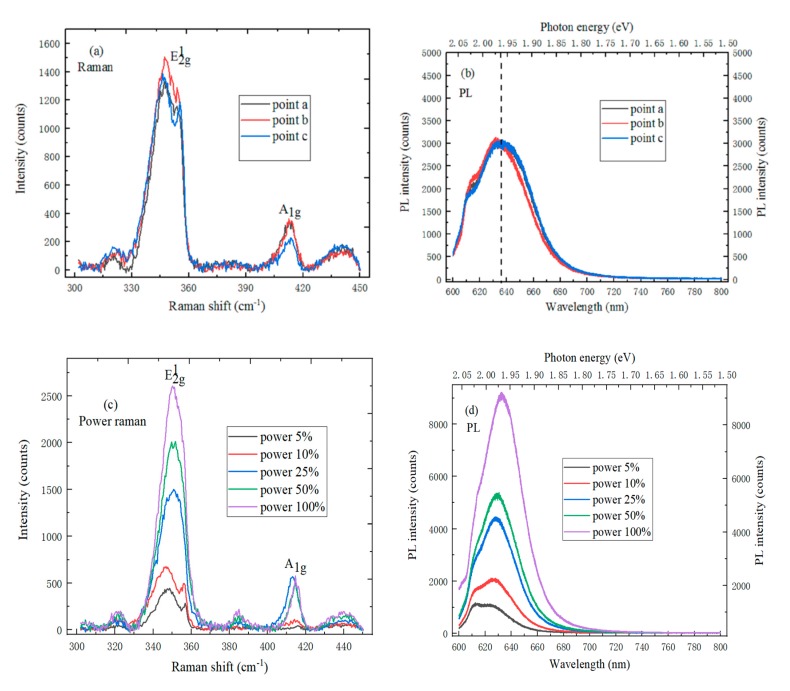
(**a**) The Raman spectrum and (**b**) Photoluminescence spectrum of monolayer WS_2_ on SiO_2_/Si substrate at three different points; (**c**) Raman spectrum and (**d**) Photoluminescence spectrum of monolayer WS_2_ on SiO_2_/Si Substrate under different laser powers.

It can be found, by observing Figure 5a, that there are two characteristic peaks in the Raman spectrum of the WS_2_ sample, and the peak positions of the E^1^_2g_ and A_1g_ characteristic peaks are respectively located at 349 cm^−1^ and 413 cm^−1^, which are fitted by the Gaussian function. The peak shift between two characteristic peaks decreases with the layer number decreases, which can be explained by the increase of the Van der Waals force. The Raman spectrums on the triangular WS_2_ sample remain consistent at different points, which can indicate that the sample is uniform. In Figure 5b, the strongest PL spectrum peak position of monolayer WS_2_ is located at 636 nm. The band gap of monolayer WS_2_ is calculated at 1.96 eV from the conversion relationship between the wavelength and electron volt, which is the same as the reported results. Besides, the PL spectrum also has an exciton peak of 2.04 eV. This is because the WS_2_ sample has energy band splitting [23]. Figure 5c shows the Raman spectrum of monolayer WS_2_ under the different laser powers. The Raman spectrum shape and relative position of the WS_2_ sample move, to a certain extent, when the laser power increases. The Raman intensity of the E^1^_2g_ characteristic peak increases with laser power increase, and the E^1^_2g_ characteristic peak shape changes, especially when the laser power exceeds 10%. Meanwhile, the A_1g_ characteristic peak intensity remains constant, but the position has a certain deviation. The PL spectrum intensity of the WS_2_ sample strongest peak increases with a laser power increase, the strongest peak position is red-shifted, and the shape changes to some extent, as shown in Figure 5d. This phenomenon can be explained from two aspects here; on the one hand, higher power would generate more heat on the WS_2_ sample, which has an effect on the power PL spectrum of WS_2_; on the other hand, the SiO_2_/Si substrate selected in the WS_2_ growth experiment is an n-type doped semiconductor material [24].

### 4.2. The Spectrum Characterization of Monolayer MoS_2_

**Figure 6:**
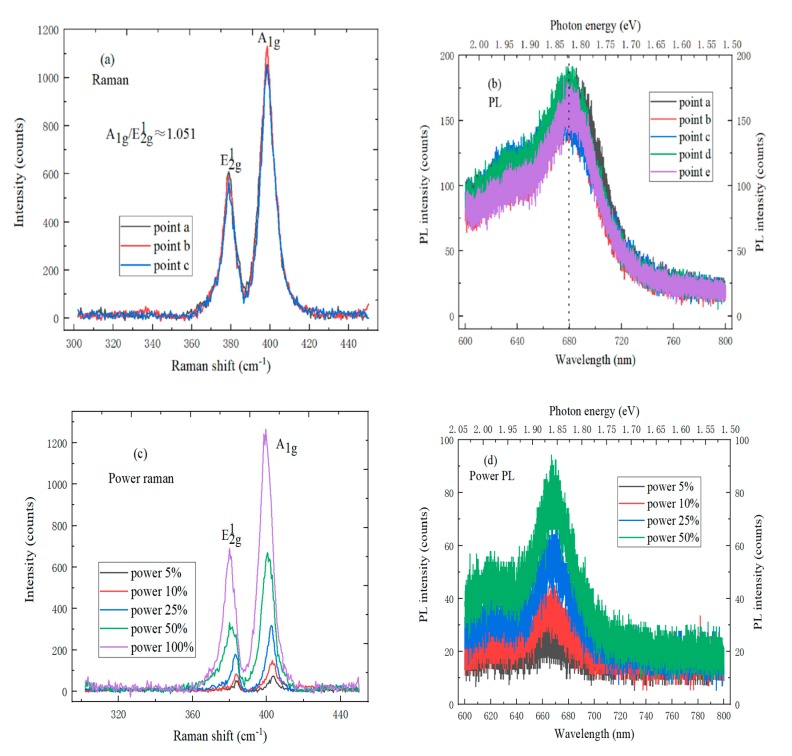
(**a**) Raman spectrum and (**b**) Photoluminescence (PL) spectrum of monolayer MoS_2_ on SiO_2_/Si substrate at different points; (**c**) Raman spectrum and (**d**) PL spectrum of monolayer MoS_2_ under different laser powers.

In Figure 6a, the Raman spectrum of monolayer MoS_2_ has two characteristic peaks, wherein the E^1^_2g_ characteristic peak is at 379 cm^−1^, the position of A_1g_ characteristic peak is 398 cm^−1^, and the ratio of A_1g_ to E^1^_2g_ characteristic peaks is approximately 1.051. Besides, the Raman spectrum intensity and position of the MoS_2_ sample at different points are consistent, which shows that the monolayer MoS_2_ sample is highly uniform. It can be seen from Figure 6b that the strongest PL spectrum peak position of monolayer MoS_2_ on SiO_2_/Si substrate is at 681.2 nm, and the electron volt is 1.82 eV, which is the same as the direct band gap width of monolayer MoS_2_. Besides, the PL spectrum of monolayer MoS_2_ also has an exciton peak at 1.95 eV. The reason is that the Mo atom has 3d orbital electron interaction [25]. As shown in Figure 6c, the Raman spectrum intensity of MoS_2_ sample increases when the laser power increases; the positions of E^1^_2g_ and A_1g_ characteristic peaks are blue-shifted simultaneously, which is caused by the n-type doped semiconductor characteristics of SiO_2_/Si substrate [26]. Figure 6d shows the PL spectrum of monolayer MoS_2_ under different laser powers. As the laser power increases, the strongest photoluminescence spectrum peak intensity of monolayer MoS_2_ increases, and the shape and position of characteristic peaks do not change. It can be judged that MoS_2_ on SiO_2_/Si substrate is monolayer, by characterizing and analyzing the Raman spectrum and photoluminescence spectrum of MoS_2_.

### 4.3. Spectrum Characterization of the MoS_2_/WS_2_ Heterojunction

**Figure 7:**
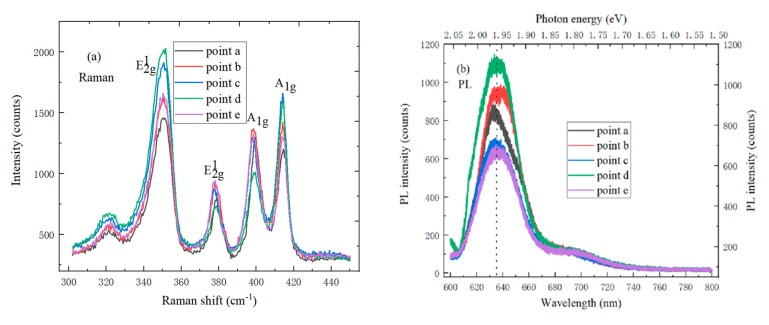

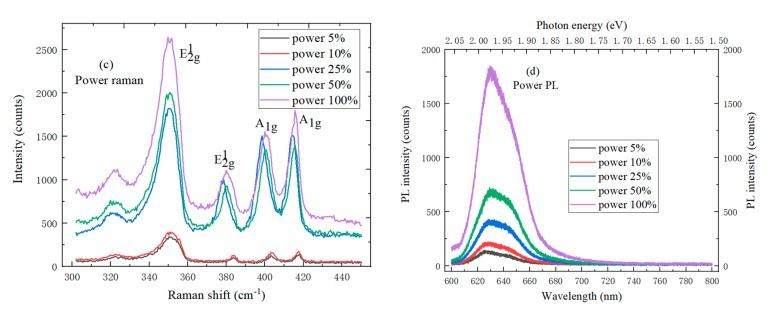
(**a**) Raman spectrum and (**b**) PL spectrum of MoS_2_/WS_2_ hetero-junction on SiO_2_/Si substrate at five points; (**c**) Power Raman spectrum and (**d**) Power photoluminescence spectrum of MoS_2_/WS_2_ hetero-junction on SiO_2_/Si substrate.

The Raman spectrum of the MoS_2_/WS_2_ hetero-junction is tested with the 532 nm laser excitation wavelength, to evaluate vibration characteristics and thickness. There are E^1^_2g_ and A_1g_ Raman active modes in the Raman spectrum of MoS_2_/WS_2_ hetero-junction, wherein the E^1^_2g_ is the in-plane optical mode that corresponds to the vibrational motion of Mo and S atoms in the x and y planes, while the A_1g_ is an out-of-plane vibration mode that corresponds to the vibrational motion of two S atoms along the z unit cell axis [27]. It can be found that four Raman characteristic peaks, WS_2_-E^1^_2g_, MoS_2_-E^1^_2g_, MoS_2_-A_1g_, and WS_2_-A_1g_, appeared in the MoS_2_/WS_2_ hetero-junction region, and the corresponding peak positions are respectively located at 350 cm^−1^, 379 cm^−1^, 398 cm^−1^ and 413 cm^−1^. In the four Raman peaks of MoS_2_/WS_2_ hetero-junction, two peaks are the same as the Raman peaks of MoS_2_ film, and the other two peaks correspond to the Raman peaks of WS_2_ film. The four Raman peaks positions of MoS_2_/WS_2_ hetero-junction did not show red or blue shift, which indicates that WS_2_ and MoS_2_ did not affect the long-distance Coulomb interaction between the effective charges [28]. The photoluminescence spectrum can be used to identify the band gaps of WS_2_ and MoS_2_. Figure 7b shows the PL spectrum of WS_2_/MoS_2_ hetero-junction under the 532 nm wavelength. The PL peak positions of WS_2_ and MoS_2_ are respectively located at 635 nm (1.96 eV) and 682 nm (1.82 eV), which is due to the direct exciton transition energy of bottom layer MoS_2_ and top layer WS_2_. It can be seen by comparing and analyzing the PL spectrum of MoS_2_/WS_2_ vertical hetero-junction and single MoS_2_ or WS_2_ thin films that the PL peak position of MoS_2_/WS_2_ hetero-junction is the same as that of the single MoS_2_ or WS_2_ films, but the peak intensity of the MoS_2_/WS_2_ hetero-junction is much lower than that of the single WS_2_ films, which can be caused by interlayer exciton relaxation. At the interface between MoS_2_ and WS_2_ composites, electrons from the conduction band of WS_2_ are transferred to the conduction band of MoS_2_, and the holes from the valence band of MoS_2_ are moved to the valence band of WS_2_, which would cause the separation of photo-generated electrons and holes [29]. A slight PL position shift and an additional weak peak of 1.82 eV can be observed in the epitaxial MoS_2_/WS_2_ hetero-junction, which can be attributed to the recombination of spatially separated carriers. The two main factors of the PL spectrum quenching are the energy and charge transfer. In Figure 7c, the four characteristic peaks intensity of MoS_2_/WS_2_ hetero-junction gradually increases with the laser power increase, wherein the E^1^_2g_ and A_1g_ characteristic peak positions of bottom layer MoS_2_ are blue-shifted, and the A_1g_ characteristic peak position of top layer WS_2_ also shifted blue. Different laser powers on MoS_2_/WS_2_ hetero-junction would generate different heat, which can affect the Raman spectrum of MoS_2_/WS_2_ hetero-junction. The top layer WS_2_ may prevent the effective collection of Raman scattered signals from the bottom layer MoS_2_. Meanwhile, the Raman signal from WS_2_ is also weakened when it passes the bottom layer of MoS_2_, indicating that the bottom layer MoS_2_ can still affect the Raman intensity of the top layer WS_2_. Figure 7d shows the photoluminescence spectrum of the MoS_2_/WS_2_ hetero-junction under different laser powers. The strongest photoluminescence peak intensity of the MoS_2_/WS_2_ hetero-junction increases with laser power increase; the strongest photoluminescence spectrum position red-shifted, and the strongest photoluminescence spectrum shape of MoS_2_/WS_2_ hetero-junction also changes.

## 5. Conclusions

The 2D TMDs materials have different energy band structures and band gaps, so that the physical properties of 2D TMDs materials can be finely adjusted by stacking with each other. The large-area, high-quality monolayer MoS_2_ and WS_2_ materials are first prepared by APCVD method in this paper. Then, the MoS_2_/WS_2_ vertical hetero-junction is prepared by the dry transfer method, which can provide a well-defined interface between WS_2_ and MoS_2_ in the vertical dimension. The interface environment is the key factor affecting the performance of VDW hetero-structure modulation devices. The optimal reaction conditions of MoS_2_/WS_2_ vertical hetero-junction by the APCVD and dry transfer methods are obtained through experiments. The composite materials are divided into the MoS_2_, WS_2_ and MoS_2_/WS_2_ hetero-junction regions, according to the morphology evolution of the MoS_2_/WS_2_ vertical hetero-junction on the SiO_2_/Si substrate. Compared to the single MoS_2_ or WS_2_ materials, the PL spectrum characterization results show that the PL intensity of the MoS_2_/WS_2_ hetero-junction is reduced by half, which is due to the effective photoelectron-hole separation phenomenon which occurred during the recombination process. Meanwhile, the strong interlayer coupling and effective interlayer charge transfer of the MoS_2_/WS_2_ hetero-junction are systematically studied to strengthen the basic understanding of interlayer coupling, which can provide an unstructured and convenient method to explore the interface environment of the VDW hetero-structures. At the same time, the characterization results can help the mass production of various VDW hetero-junctions in future optoelectronic devices.
